# A multistrain probiotic increases the serum glutamine/glutamate ratio in patients with cirrhosis: a metabolomic analysis

**DOI:** 10.1097/HC9.0000000000000072

**Published:** 2023-04-04

**Authors:** Luca Laghi, Eva Román, Qiuyu Lan, Juan Camilo Nieto, Aleix Canalda-Baltrons, Maria Poca, Maria B. Sánchez-Rodríguez, Joan Clària, Edilmar Alvarado, Berta Cuyàs, Elisabet Sánchez, Sílvia Vidal, Carlos Guarner, Àngels Escorsell, Chaysavanh Manichanh, German Soriano

**Affiliations:** 1 Department of Agricultural and Food Sciences, University of Bologna, Cesena, Italy; 2 Department of Gastroenterology, Hospital de la Santa Creu i Sant Pau, Universitat Autònoma de Barcelona, Barcelona, Spain; 3 Escola Universitària d’Infermeria EUI-Sant Pau, Hospital de la Santa Creu i Sant Pau, Universitat Autònoma de Barcelona, Barcelona, Spain; 4 CIBERehd, Instituto de Salud Carlos III, Madrid, Spain; 5 Institut de Recerca IIB-Sant Pau, Hospital de la Santa Creu i Sant Pau, Universitat Autònoma de Barcelona, Barcelona, Spain; 6 Vall d’Hebron Institut de Recerca (VHIR), Barcelona, Spain; 7 Hospital Clínic-Institut d’Investigacions Biomèdiques August Pi i Sunyer (IDIBAPS), Universitat de Barcelona, Barcelona, Spain; 8 European Foundation for the Study of Chronic Liver Failure (EF Clif), Barcelona, Spain; 9 Department of Immunology, Hospital de la Santa Creu i Sant Pau, Universitat Autònoma de Barcelona, Barcelona, Spain

## Abstract

To explore the potential mechanisms underlying the effects of a probiotic in cirrhotic patients, we analyzed the blood metabolome using proton nuclear magnetic resonance (^1^H-NMR) spectroscopy in 32 patients with cirrhosis and cognitive dysfunction or falls. Patients were randomized to receive a multistrain probiotic or placebo for 12 weeks. Among the 54 metabolites identified, the only significant changes in the probiotic group were an increase in glutamine, a decrease in glutamate, and an increase in the glutamine/glutamate ratio. In the placebo group, glutamate increased and the glutamine/glutamate ratio decreased. Our results suggest the multistrain probiotic could influence glutamine/glutamate metabolism, increasing the capacity of ammonia detoxification.

In a previous double-blinded, placebo-controlled, randomized clinical trial, we observed that a multistrain probiotic improved cognitive function and risk of falls, reinforced the intestinal barrier, and modulated the proinflammatory state in patients with cirrhosis.^1^ However, the mechanisms underlying these effects are not well known. One approach that can help identify such mechanisms is metabolomics.

We analyzed serum metabolome in samples from 32 outpatients with cirrhosis included in the study mentioned above.^1^ All patients had cognitive dysfunction (psychometric hepatic encephalopathy score [PHES] <−4) and/or falls during the previous year. Seventeen patients were treated for 12 weeks with the multistrain probiotic De Simone Formulation (Vivomixx) 450×10^9^ cfu bid for 12 weeks, and 15 were treated with placebo. We performed untargeted metabolomics by proton nuclear magnetic resonance (^1^H-NMR) using a procedure previously described.^2^ A subgroup of 9 patients in the probiotic group and 6 in the placebo group also provided fecal samples for the study of microbiota composition using 16S rRNA gene sequencing.^1^

Patients had a mean age of 64.5 years, 65.6% were women, the etiology of cirrhosis was alcohol in 50%, the mean model for end-stage liver disease (MELD) score was 9.3, 81.2% had previous decompensations, 84.3% had previous falls, and 28.1% presented a PHES <−4. There were no statistical differences between the 2 groups at baseline.

The untargeted observation of the serum metabolome by ^1^H-NMR allowed the unambiguous assignment of 54 metabolites (Supplemental Fig. 1, http://links.lww.com/HC9/A166, and Supplemental Table 1, http://links.lww.com/HC9/A167). Pairwise comparisons between values at baseline and at 12 weeks (Figure 1) showed blood glutamine increased in the probiotic group (*p* = 0.002, FDR *p* = 0.007) while glutamate decreased (*p* = 0.03, FDR *p* = 0.03), leading to an increase in the glutamine/glutamate ratio (*p* = 0.009, FDR *p* = 0.01). In contrast, the placebo group showed an increase in glutamate concentration (*p* = 0.01, FDR *p* = 0.02) and a decrease of the glutamine/glutamate ratio (*p* = 0.02, FDR *p* = 0.03). No statistically significant changes were observed in any of the other metabolites identified. We found a correlation between the change in the glutamine/glutamate ratio and the change in the PHES (*r* = 0.34, *p* = 0.05), the change in the risk of falls evaluated by the gait speed (*r* = 0.47, *p* = 0.008), and the change in the intestinal barrier integrity assessed by the fatty-acid binding protein 6 (FABP-6) (*r* = −0.37, *p* = 0.04). We evaluated mitochondrial dysfunction by determining serum fibroblast growth factor 21 (FGF-21), but we did not find a correlation between the change in FGF-21 and the change in glutamine or the change in the glutamine/glutamate ratio.

**FIGURE 1:**
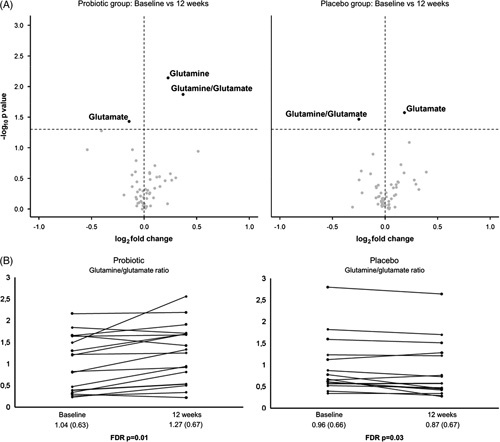
(A) Volcano plots showing the change between baseline and 12 weeks in all metabolites identified in the probiotic group and in the placebo group. (B) Changes in glutamine/glutamate ratio in patients treated with the probiotic and patients treated with placebo. Results are expressed as mean (SD). Abbreviation: FDR, false discovery rate.

Regarding metagenomic results, when we focused on the serum glutamine/glutamate ratio, analyzing all the 30 samples at the genus level, we observed this ratio was associated with an abundance of *Paraprevotella* and *Oscillospira* in feces (coefficient: −0.53, *p* = 0.002, *q* = 0.07, and coefficient: −0.29, *p* = 0.02, *q* = 0.21, respectively). The abundance of *Oscillospira* was also associated with gait speed (coefficient: −1.05, *p* = 0.01, *q* = 0.15).

The increase in the serum glutamine/glutamate ratio observed in the present study in probiotic-treated patients could reflect a higher capacity of ammonia clearance because ammonia binds to glutamate to generate glutamine.^3^ Unfortunately, we did not measure serum ammonia levels, but other authors have demonstrated a decrease in ammonemia associated with a decrease in hepatic encephalopathy (HE) episodes in patients with cirrhosis treated with this probiotic formulation.^4^ A higher ammonia clearance capacity and the subsequent decrease in ammonemia, together with a decrease in the proinflammatory state, would explain the improvement in cognitive function and risk of falls observed in patients treated with the probiotic.^1^ This hypothesis is further supported by the positive correlation between the change in the glutamine/glutamate ratio and cognitive function, and the negative correlation between the ratio and the risk of falls.

Our findings are in line with a recent study in rats with bile duct ligation, which showed the multistrain probiotic used here ameliorated locomotor activity and improved the neurometabolic profile assessed by in vivo brain ultrahigh field ^1^H-NMR spectroscopy.^5^ The authors found an attenuation in the increase in brain glutamine and a lesser decrease in myo-inositol and glutamate. These changes indicate a reversal of the characteristic neurometabolic alterations observed in hyperammonemia and HE.

The exact mechanisms underlying the increase in the glutamine/glutamate ratio cannot be elucidated from the present study. One possible mechanism is that the probiotic could induce a decrease in glutaminase activity or an increase in glutamine synthetase activity, as has been observed in experimental models with other treatments for HE, such as rifaximin^6^ or ornithine phenylacetate.^7^ Another potential explanation for the increase in the glutamine/glutamate ratio is an improvement in mitochondrial dysfunction. Mitochondrial dysfunction can lead to a decrease in glutamine due to its utilization to fuel the mitochondria through anaplerotic reactions to obtain energy.^8^ However, the lack of correlation between the change in the glutamine/glutamate ratio and the change in FGF-21 does not support this hypothesis.

Regarding gut microbiota composition, with the limitations of the small sample size, here we focused on the glutamine/glutamate ratio and found that higher values in blood were associated with lower abundance of *Paraprevotella* and *Oscillospira* in feces. We failed to find a relationship between these bacteria and ammonia metabolism in previous literature. However, the abundance of *Paraprevotella* has been associated with sedentarism,^9^ and the abundance of *Oscillospira* with physical frailty and sarcopenia in older people.^10^ In agreement with these data, we observed a negative association between the abundance of *Oscillospira* and gait speed—a parameter related to frailty.^1^ Moreover, the change in gait speed correlated positively with the change in the glutamine/glutamate ratio. These data suggest a possible relationship between *Oscillospira* abundance in feces, frailty and ammonia metabolism in patients with cirrhosis.

In conclusion, metabolomic analysis in patients with cirrhosis treated with this multistrain probiotic showed an increase in the serum glutamine/glutamate ratio. This finding contributes to the understanding of the mechanisms involved in the effects of this probiotic and could help in the design of new approaches to improve patients’ outcomes and health-related quality of life.

## Supplementary Material
