# Supplementary figures and images for: A multistrain probiotic increases the serum glutamine/glutamate ratio in patients with cirrhosis: a metabolomic analysis
Source: Hepatol Commun. 2023 Apr 4;7(4):e0072. doi: 10.1097/HC9.0000000000000072 (PMC10079330; doi:10.1097/HC9.0000000000000072)

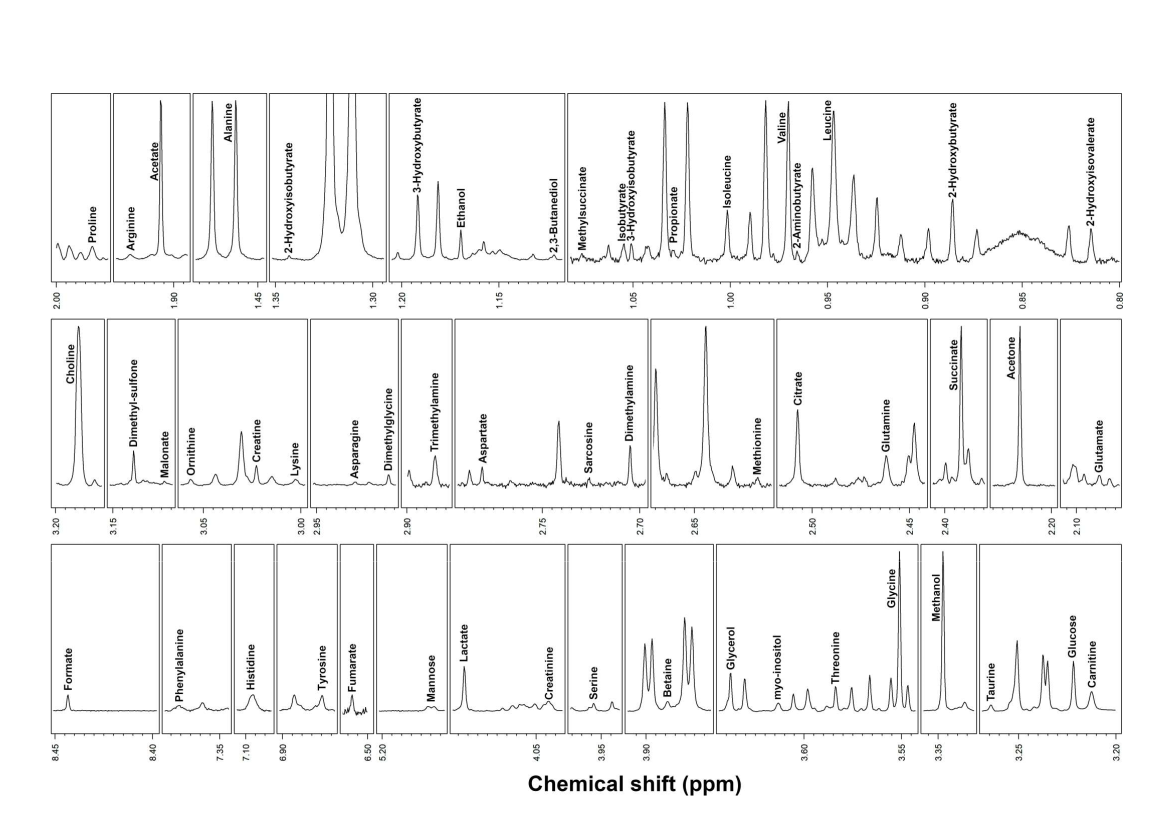

Supplement: Supplementary file 1 [file hc9-7-e0072-s001.tiff]
