# Supplementary material for: A multistrain probiotic increases the serum glutamine/glutamate ratio in patients with cirrhosis: a metabolomic analysis
Source: Hepatol Commun. 2023 Apr 4;7(4):e0072. doi: 10.1097/HC9.0000000000000072 (PMC10079330; doi:10.1097/HC9.0000000000000072)
Supplement: Supplementary file 2 [file hc9-7-e0072-s002.docx]

**SUPPLEMENTARY TABLES**

**Supplementary Table 1.** Molecules of serum that did not show significant differences between baseline and after treatment. For each molecule, the p value is reported for patients treated with placebo and for patients treated with the probiotic formulation.

|  | Placebo | Probiotic |  |  | Placebo | Probiotic |
| --- | --- | --- | --- | --- | --- | --- |
| Acetone | 0.93 | 0.053 |  | Alanine | 0.63 | 0.54 |
| Dimethylglycine | 0.60 | 0.11 |  | Tyrosine | 0.66 | 0.55 |
| 3-Hydroxybutyrate | 0.39 | 0.11 |  | Fumarate | 0.74 | 0.56 |
| 2,3-Butanediol | 0.33 | 0.11 |  | 2-Hydroxyisobutyrate | 0.54 | 0.59 |
| Betaine | 0.27 | 0.16 |  | Threonine | 0.99 | 0.61 |
| Methionine | 0.45 | 0.20 |  | Acetate | 0.19 | 0.64 |
| Arginine | 0.74 | 0.25 |  | Dimethyl-sulfone | 0.95 | 0.64 |
| Taurine | 0.34 | 0.25 |  | Creatinine | 0.68 | 0.66 |
| Formate | 0.96 | 0.26 |  | Sarcosine | 0.58 | 0.67 |
| Ethanol | 0.77 | 0.26 |  | 2-Hydroxybutyrate | 0.48 | 0.69 |
| Mannose | 0.62 | 0.28 |  | Trimethylamine | 0.36 | 0.75 |
| Carnitine | 0.08 | 0.30 |  | Choline | 0.25 | 0.78 |
| Methanol | 0.40 | 0.30 |  | Leucine | 0.80 | 0.80 |
| Malonate | 0.83 | 0.31 |  | Ornithine | 0.66 | 0.80 |
| 3-Hydroxyisobutyrate | 0.63 | 0.33 |  | Phenylalanine | 0.85 | 0.82 |
| Glycerol | 0.40 | 0.34 |  | Dimethylamine | 0.43 | 0.82 |
| myo-Inositol | 0.54 | 0.34 |  | Isobutyrate | 0.83 | 0.82 |
| Isoleucine | 0.55 | 0.36 |  | Proline | 0.67 | 0.89 |
| Lactate | 0.89 | 0.43 |  | 2-Hydroxyisovalerate | 0.93 | 0.89 |
| 2-Aminobutyrate | 0.44 | 0.43 |  | Glycine | 0.45 | 0.89 |
| Creatine | 0.88 | 0.46 |  | Methylsuccinate | 0.26 | 0.89 |
| Asparagine | 0.86 | 0.48 |  | Aspartate | 0.80 | 0.91 |
| Valine | 0.87 | 0.48 |  | Citrate | 0.87 | 0.92 |
| Lysine | 0.13 | 0.49 |  | Propionate | 0.91 | 0.95 |
| Glucose | 0.24 | 0.50 |  | Succinate | 0.37 | 0.97 |
| Histidine | 0.71 | 0.52 |  | Serine | 0.75 | 0.99 |

**SUPPLEMENTARY FIGURE LEGENDS**

**Supplementary Figure 1.** Parts of spectra obtained by ^1^H-NMR, representative of all spectra obtained in the study. The name of each molecule is shown above the NMR signal used for its quantification. To ease the visual inspection of each portion, we selected a different vertical magnification.
